# A Noncontact FMCW Radar Sensor for Displacement Measurement in Structural Health Monitoring

**DOI:** 10.3390/s150407412

**Published:** 2015-03-26

**Authors:** Cunlong Li, Weimin Chen, Gang Liu, Rong Yan, Hengyi Xu, Yi Qi

**Affiliations:** 1 College of Optoelectronic Engineering, Chongqing University, Chongqing 400044, China; E-Mails: licunlongcqu@126.com (C.L.); yanrongcqu@126.com (R.Y.); hyxu0802@cqu.edu.cn (H.X.); cqu_yiqi@cqu.edu.cn (Y.Q.); 2 Key Laboratory for Optoelectronic Technology and Systems, Ministry of Education, Chongqing University, Chongqing 400044, China; 3 School of Civil Engineering, Chongqing University, Chongqing 400045, China; E-Mail: gliu@cqu.edu.cn; 4 Key Laboratory of New Technology for Construction of Cities in Mountain Area, Ministry of Education, Chongqing University, Chongqing 400045, China

**Keywords:** structural health monitoring, displacement, FMCW radar, multiple targets

## Abstract

This paper investigates the Frequency Modulation Continuous Wave (FMCW) radar sensor for multi-target displacement measurement in Structural Health Monitoring (SHM). The principle of three-dimensional (3-D) displacement measurement of civil infrastructures is analyzed. The requirements of high-accuracy displacement and multi-target identification for the measuring sensors are discussed. The fundamental measuring principle of FMCW radar is presented with rigorous mathematical formulas, and further the multiple-target displacement measurement is analyzed and simulated. In addition, a FMCW radar prototype is designed and fabricated based on an off-the-shelf radar frontend and data acquisition (DAQ) card, and the displacement error induced by phase asynchronism is analyzed. The conducted outdoor experiments verify the feasibility of this sensing method applied to multi-target displacement measurement, and experimental results show that three targets located at different distances can be distinguished simultaneously with millimeter level accuracy.

## 1. Introduction

Large infrastructures, such as high-rise buildings and long-span bridges, inevitably deteriorate under operational loading and environmental conditions, and structural failure may result in serious economic, performance, and life-safety consequences [1,2]. Thus, appropriate monitoring of these infrastructures is critical to ensure the reliability of structures and safety of human lives. Among all the available monitoring parameters of infrastructures, the structural displacements usually applied for the monitoring of deformation and vibration play an important role in Structural Health Monitoring (SHM) [3,4,5,6,7,8].

Displacement monitoring can be implemented by either contacting or non-contacting sensors. The contacting sensors include Linear Variable Differential Transformers (LVDT), dial gauges, strain gauges, and other displacement meters [5,9,10], but contacting sensors have to be mounted directly onto the measuring points of structures and be connected to a neighboring stationary reference point. For practical infrastructures, it is difficult to find an ideal stationary reference point near the measuring point, and it is also inconvenient for the connection between sensors and stationary reference points.

Without being connected to a stationary reference point directly, non-contacting sensors can be located outside the infrastructures, which is quite convenient for SHM. Non-contacting sensors used in SHM usually include total station, vision-based systems, Global Position System (GPS), and radar-based systems [11,12,13,14,15,16]. Having a millimeter measuring accuracy, total station is a fine optic-electronic instrument when operating in a good conditions. However, dust, fog and rain are harmful to its precise opto-mechanotronics when it is working in bad weather conditions [12,16]. This means that a total station is not suitable for long-term application in infrastructural sites. Vision-based systems suffer from the same problems as total stations [11,13].

Despite suitability for long-term operation in bad weather, GPS has an accuracy of meters which is insufficient for most SHM applications. Differential GPS has a highest accuracy of centimeters, but it is also insufficient and too expensive for SHM systems [14]. Radar-based systems, which are suitable for bad weather conditions as well, have been reported in many displacement measurement applications [17,18,19,20,21]. In recent years, the research interest in radar-based systems for SHM is also increasing [6,7,15], and further exploitation is expected for multi-target identification and high-accuracy displacement measurement. Although the Frequency Modulation Continuous Wave (FMCW) technique can be used to distinguish different targets by beat frequency, its measurement accuracy is limited by the frequency estimation. The phase of beat signal is introduced for upgrading the measurement accuracy, and the reported accuracy is hundreds of micrometers for one target at a distance of several meters as shown in Table 1 [22,23,24,25], which is insufficient for SHM. Therefore, further development of the radar sensors is expected in multi-target identification and high-accuracy displacement measurement.

In this paper, a preliminary study of a FMCW radar sensor for high-accuracy displacement measurement of multiple targets is presented. The three dimensional (3-D) displacement of the targets on civil infrastructures is described and the measuring principle of high-accuracy 3-D displacement is discussed. The principles of FMCW radar for high-accuracy displacement measurement and targets identification are presented. To verify the feasibility of the proposed sensing method, an off-the-shelf radar frontend and Data Acquisition (DAQ) card are employed in a FMCW radar prototype. Outdoor experiments with the radar prototype are carried out for one target and three targets respectively, and the feasibility of the sensing method using FMCW radar for multi-target displacement is confirmed.

The remainder of this paper is organized as follows: in Section 2, 3-D displacement measurement in SHM is analyzed, and requirements for the measuring sensor are discussed. Section 3 presents the measurement principle of FMCW radar for one target using beat frequency and phase estimation. In Section 4, the multi-target displacement measurement is discussed and simulated. In Section 5, the developed radar prototype is given and displacement error induced by phase asynchronism is analyzed. Section 6 describes outdoor experiments for one target and three targets, respectively. Then, the experimental results are discussed. Finally, Section 7 concludes the study.

**Table 1:** Reported accuracy of FMCW radar based on the phase of beat signal.

Reference	Distance	Accuracy
[22]	<1 m	1 mm
[23]	2 m	150 μm
[24]	2 m	75 μm
[25]	<5 m	<1 mm

## 2. Description of Displacement Measurement in SHM

### 2.1. Description of Displacement Measurement in a Large Infrastructure

**Figure 1:**
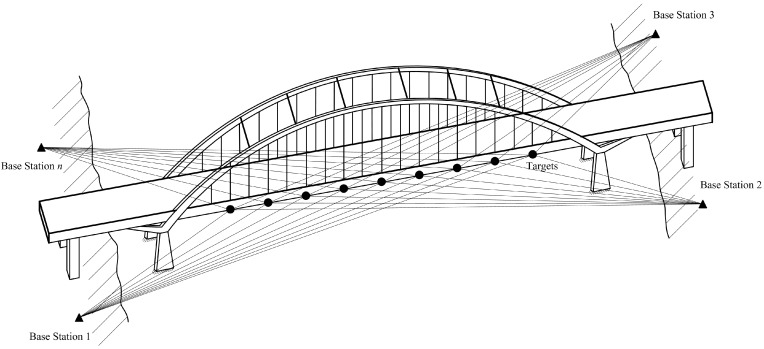
Displacement monitoring scheme of an arch-bridge.

Take an arch-bridge as an example. The displacement monitoring scheme with noncontact sensors can be depicted as shown in Figure 1. It is obvious that the deformation of the bridge must be monitored at many positions along the girder axis from different base stations.

To obtain the displacement information of each position, one target must be fixed at each monitoring position of the bridge. In addition, one measuring machine located at the base station measures the distance between the base station and each target. In order to measure the three-dimensional (3-D) displacement of each target, more than three measuring machines located at different base stations are needed.

### 2.2. 3-D Displacement Measuring Principle of Single Target

**Figure 2:**
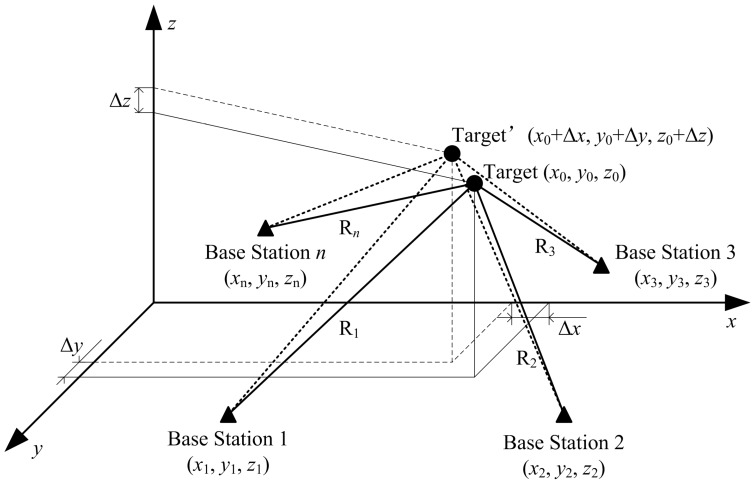
Measuring diagram of 3-D displacement based on multiple base stations.

If only one target in Figure 1 is taken into account, the target displacement can be depicted as shown in Figure 2. Supposing that there are *n* reference points: from B_1_ (*x*_1_, *y*_1_, *z*_1_), B_2_ (*x*_2_, *y*_2_, *z*_2_) to B*_n_* (*x_n_*, *y_n_*, *z_n_*), and initial location of target is A_0_ (*x*_0_, *y*_0_, *z*_0_), the distance *R_i_* between reference point B*_i_* to target A_0_ is the following:

(1) $$\left\{ \begin{array}{c} R_{1}=\sqrt{{\left(x_{0}-x_{1}\right)}^{2}+{\left(y_{0}-y_{1}\right)}^{2}+{\left(z_{0}-z_{1}\right)}^{2}}\\ R_{2}=\sqrt{{\left(x_{0}-x_{2}\right)}^{2}+{\left(y_{0}-y_{2}\right)}^{2}+{\left(z_{0}-z_{2}\right)}^{2}}\\ \vdots \\ R_{n}=\sqrt{{\left(x_{0}-x_{n}\right)}^{2}+{\left(y_{0}-y_{n}\right)}^{2}+{\left(z_{0}-z_{n}\right)}^{2}} \end{array}\right.$$

When the target moves from A_0_ (*x*_0_, *y*_0_, *z*_0_) to A (*x*_0_ + Δ*x*, *y*_0_ + Δ*y*, *z*_0_ + Δ*z*), there must be a spatial displacement $P=\vec{A_{0}A}$. This spatial displacement *P* can be divided into three components Δ*x*, Δ*y* and Δ*z* in Cartesian coordinates:

(2) $$\left\{ \begin{array}{l} \Delta x=P\cdot \cos\alpha \\ \Delta y=P\cdot \cos\beta \\ \Delta z=P\cdot \cos\gamma \end{array}\right.$$

where α, β and γ are the angles between spatial displacement *P* and the coordinate axis, respectively.

Due to displacement *P*, the distance between B*_i_* and A also changes from *R_i_* into *R_i_* + Δ*R_i_*, and Equation (1) should be rewritten as follows:

(3) $$\left\{ \begin{array}{c} R_{1}+\Delta R_{1}=\sqrt{{\left[\left(x_{0}+\Delta x\right)-x_{1}\right]}^{2}+{\left[\left(y_{0}+\Delta y\right)-y_{1}\right]}^{2}+{\left[\left(z_{0}+\Delta z\right)-z_{1}\right]}^{2}}\\ R_{2}+\Delta R_{2}=\sqrt{{\left[\left(x_{0}+\Delta x\right)-x_{2}\right]}^{2}+{\left[\left(y_{0}+\Delta y\right)-y_{2}\right]}^{2}+{\left[\left(z_{0}+\Delta z\right)-z_{2}\right]}^{2}}\\ \vdots \\ R_{n}+\Delta R_{n}=\sqrt{{\left[\left(x_{0}+\Delta x\right)-x_{n}\right]}^{2}+{\left[\left(y_{0}+\Delta y\right)-y_{n}\right]}^{2}+{\left[\left(z_{0}+\Delta z\right)-z_{n}\right]}^{2}} \end{array}\right.$$

Since *R_i_*, A_0_ (*x*_0_, *y*_0_, *z*_0_) and B*_i_* (*x_i_*, *y_i_*, *z_i_*) are determinate, the displacements Δ*x*, Δ*y*, and Δ*z* can be calculated according to Equations (1)–(3). It should be noted that Equation (3) is nonlinear, so the exact analytic solution cannot be obtained unless processed linearly. Compared with the distance *R_i_*, both distance change and displacement are very small, thus Equation (3) could be approximated into a Taylor expansion as follows:

(4) $$R_{i}+\Delta R_{i}=R_{i}+\frac{\left(x_{0}-x_{i}\right)}{R_{i}}\Delta x+\frac{\left(y_{0}-y_{i}\right)}{R_{i}}\Delta y+\frac{\left(z_{0}-z_{i}\right)}{R_{i}}\Delta z$$

where $R_{i}=\sqrt{{\left(x_{0}-x_{i}\right)}^{2}+{\left(y_{0}-y_{i}\right)}^{2}+{\left(z_{0}-z_{i}\right)}^{2}}$.

According to matrix $\vec{\Delta P}={\left(\Delta x,\;\Delta y,\;\Delta z\right)}^{T}$ and matrix $\vec{\Delta R}={\left(\Delta R_{1},\;\Delta R_{2},\;\cdots \Delta R_{n}\right)}^{T}$, the linear expression of Equation (3) can be expressed as:

(5) $$\vec{\Delta R}=\vec{M}\cdot \vec{\Delta P}$$

where: $\vec{M}=\left(\begin{array}{ccc} \frac{x_{0}-x_{1}}{R_{1}} & \frac{y_{0}-y_{1}}{R_{1}} & \frac{z_{0}-z_{1}}{R_{1}}\\ \frac{x_{0}-x_{2}}{R_{2}} & \frac{y_{0}-y_{2}}{R_{2}} & \frac{z_{0}-z_{2}}{R_{2}}\\ \vdots & \vdots & \vdots \\ \frac{x_{0}-x_{n}}{R_{n}} & \frac{y_{0}-y_{n}}{R_{n}} & \frac{z_{0}-z_{n}}{R_{n}} \end{array}\right)$.

Based on the least square method, the 3-D displacement (Δ*x*, Δ*y*, Δ*z*) of the target can be obtained by:

(6) $$\vec{\Delta P}={\left({\vec{M}}^{T}\cdot \vec{M}\right)}^{-1}\cdot {\vec{M}}^{T}\cdot \vec{\Delta R}$$

Since all elements in matrix $\vec{M}$ are fixed, $\vec{\Delta P}$ can be determined by $\vec{\Delta R}$, and thus 3-D displacement (Δ*x*, Δ*y*, Δ*z*) can be gotten by the distance change $\vec{\Delta R}$. It is obvious that measurement of distance change Δ*R_i_* is the key for 3-D displacement measurement. We can get the measuring error by differentiating Equation (4), as follows:

(7) $$\sigma_{\Delta R_{i}}=\frac{1}{R_{i}}\sqrt{{\left(x_{0}-x_{i}\right)}^{2}\cdot {\sigma_{\Delta x}}^{2}+{\left(y_{0}-y_{i}\right)}^{2}\cdot {\sigma_{\Delta y}}^{2}+{\left(z_{0}-z_{i}\right)}^{2}\cdot {\sigma_{\Delta z}}^{2}}$$

where $R_{i}=\sqrt{{\left(x_{0}-x_{i}\right)}^{2}+{\left(y_{0}-y_{i}\right)}^{2}+{\left(z_{0}-z_{i}\right)}^{2}}$.

Supposing that $\sigma_{\Delta x}=\sigma_{\Delta y}=\sigma_{\Delta z}$, Equation (7) can be rewritten as:

(8) $$\sigma_{\Delta R_{i}}=\sigma_{\Delta x}$$

According to Equation (8), it can be concluded that the accuracy of displacement $\vec{\Delta P}$ is proportional to the distance change $\vec{\Delta R}$. It means that high-accuracy measurement of the distance change is necessary in spatial displacement measurement of SHM.

Although the above discussion concerns a single target, it is still suitable for the multiple targets shown in Figure 1. When there are several targets installed in the infrastructure, multi-target identification is needed to demodulate the corresponding distance change of each target. Therefore, high-accuracy measurement of distance change and multi-target identification are the two key factors for the sensors applied in SHM.

## 3. FMCW Radar Sensor for Displacement Measurement of Single Target

### 3.1. Signal Propagation of FMCW Radar

**Figure 3:**
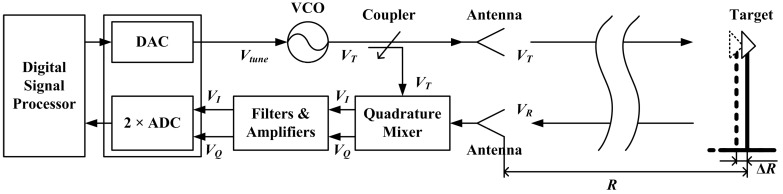
Schematic diagram of a FMCW radar sensor.

To address the problems of high-accuracy distance change (displacement) measurement and multi-target identification, the FMCW radar is introduced for SHM. Although a FMCW radar will be required in each base station for 3-D displacement measurements, all of the FMCW radars used are the same. The overall diagram of the FMCW radar sensor is shown in Figure 3. The Digital-to-Analog Converter (DAC) is controlled by Digital Signal Processor (DSP) to generate a triangle wave or sawtooth wave used for controlling a Voltage Controlled Oscillator (VCO). The VCO generates a microwave signal *V_T_* whose frequency changes along with time. The coupler divides the microwave signal *V_T_* into two parts, one is transmitted towards the target while the other is used as the replica of the transmitted signal. The reflected signal *V_R_* is received by the antenna and then fed into the Quadrature Mixer where *V_R_* is mixed with the replica of *V_T_* to generate In-phase/Quadrature (I/Q) signals. After signal-conditioning, I/Q signals are fed into the Analog-to-Digital Converter (ADC) ports. After the data acquisition by ADC, sampled signals *V_I_* and *V_Q_* are processed in the DSP. Finally, the distance *R* and displacement Δ*R* of the target are calculated and stored in DSP.

**Figure 4:**
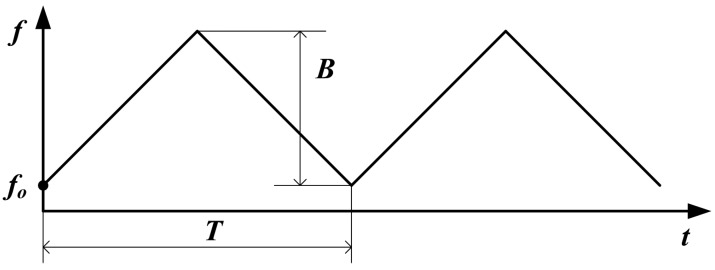
Instantaneous frequency of the transmitted microwave.

Supposing that the VCO is controlled by a triangle wave (applied in our sensor), the frequency of the transmitted microwave is also the triangle wave shown in Figure 4. In the valid duration of upchirp, it can be expressed by:

(9) $$\begin{array}{cc} f\left(t\right)=f_{o}+2B/T\cdot t, & 0\leq t<T/2 \end{array}$$

where *f_o_* is the initial frequency, *T*/2 is the duration of upchirp, and *B* is the bandwidth of transmitted microwave [25,26]. The instantaneous phase of the transmitted signal can be obtained by calculating the integral of Equation (9):

(10) $$\Phi_{T}\left(t\right)=\Phi_{o}+2\pi \int_{0}^{t}f\left(t\right)dt=\Phi_{o}+2\pi f_{o}\cdot t+2\pi B/T\cdot t^{2},0\leq t<T/2$$

where Φ*_o_* is the initial phase, and the transmitted signal can be expressed by:

(11) $$\begin{array}{cc} V_{T}\left(t\right)=e^{j\left(\Phi_{o}+2\pi f_{o}\cdot t+2\pi B/T\cdot t^{2}\right)}, & 0\leq t<T/2 \end{array}$$

Assuming that the distance from one stationary target to the sensor is presented by *R*, the received signal reflected from the target is given by:

(12) $$\begin{array}{cc} V_{R}\left(t\right)=Ae^{j\left[\Phi_{o}+2\pi f_{o}\cdot \left(t-\tau \right)+2\pi B/T\cdot {\left(t-\tau \right)}^{2}\right]}, & 0\leq t<T/2 \end{array}$$

where *A* is signal amplitude (dependent on the Radar Cross Section of target, transmitted power, *etc.*), τ = 2*R*/*c* is the round-trip time delay from transmitter to receiver and *c* is the propagation velocity of electromagnetic wave [25,26]. The received signal is mixed with the replica of transmitted signal in the quadrature mixer as shown in Figure 3. After demodulation, I/Q signals are obtained, which comprise the beat signal *v_b_*:

(13) $$\left\{ \begin{array}{l} \begin{array}{cc} V_{I}=A\cos\left(4\pi B\tau /T\cdot t+2\pi f_{o}\cdot \tau -2\pi B/T\cdot \tau^{2}\right), & 0\leq t<T/2 \end{array}\\ \begin{array}{cc} V_{Q}=A\sin\left(4\pi B\tau /T\cdot t+2\pi f_{o}\cdot \tau -2\pi B/T\cdot \tau^{2}\right), & 0\leq t<T/2 \end{array} \end{array}\right.$$

The complex expression *v_b_*(*t*) is created:

(14) $$\begin{array}{cc} v_{b}\left(t\right)=V_{I}+jV_{Q}=Ae^{j\left(4\pi B\tau /T\cdot t+2\pi f_{o}\cdot \tau -2\pi B/T\cdot \tau^{2}\right)}, & 0\leq t<T/2 \end{array}$$

Notice that τ is negligible compared with the valid duration *T*/2, so the third term of Equation (14) can be ignored. We can obtain the frequency *f_b_* and phase Φ*_b_* of the beat signal by the Fourier Transform spectral analysis technique. It should be noticed that the time delay *τ* is not only included in the frequency of the beat signal, but also in the phase. Therefore, the distance *R* could be determined by *f_b_* and Φ*_b_*, respectively.

### 3.2. Displacement Measurement of Single Target

To demodulate the frequency and phase of beat signal from Equation (14), the continuous signal should be sampled first, and then *f_b_* and Φ*_b_* of the beat signal are calculated by Discrete Fourier Transform (DFT) of the sampled signal. The sampled beat signal can be written as:

(15) $$\begin{array}{cc} v_{bD}\left(n\right)=Ae^{j\left(4\pi B\tau /N\cdot n+2\pi f_{o}\cdot \tau \right)}, & 0\leq n<N/2 \end{array}$$

where *n* is the discrete time index, *N* is the number of recorded samples during one period *T*, and the third term of Equation (14) is ignored. We can obtain the spectrum of Equation (15) by DFT:

(16) $$\begin{array}{ll} V_{B}\left(k\right) & =\sum_{n=0}^{N/2-1}v_{bD}\left(n\right)\cdot \exp\left(-j\frac{2\pi }{N/2}nk\right)\\ & \begin{array}{cc} =A\exp j\left[2\pi \left(f_{0}+\frac{N-2}{N}\frac{B}{2}\right)\tau -\pi \frac{N-2}{N}k\right]\cdot \frac{\sin\left[\pi \left(B\tau -k\right)\right]}{\sin\left[\frac{\pi }{N/2}\left(B\tau -k\right)\right]}, & 0\leq k<N/2 \end{array} \end{array}$$

Then, we can obtain the beat-frequency bin *k_b_* by searching the maximum of Equation (16). Due to the finite length of Equation (15), the frequency resolution of spectrum is 2/*T*, and the demodulated beat frequency from Equation (16) will be [27]:

(17) $$f_{b}=k_{b}\frac{2}{T}$$

The distance calculated by frequency will also be discrete. According to *k_b_* = *B*τ and *R* = *c*τ/2, the range resolution is:

(18) $$\Delta R_{res}=c/2B$$

Finally, the target distance *R* can be calculated by:

(19) $$R=k_{b}\cdot \Delta R_{res}$$

However, the range resolution is constrained by the microwave bandwidth *B*, and the distance measurement will also be affected by the nonlinearity of transmitted microwave. Therefore, the target displacement based on Equation (19) will be inaccurate. Considering that the reported displacement of civil infrastructure is usually less than dozens of centimeters, so the fixed target on the structure moves in a small region. Therefore, if the range resolution of FMCW radar is at the level of meters, the fixed target will always be in the same range bin. Under this circumstance, the phase of the beat signal can always be obtained from the fixed frequency bin *k_b_* of Equation (16), and the phase Φ*_b_* is:

(20) $$\Phi_{b}=2\pi \left(f_{0}+\frac{N-2}{N}\frac{B}{2}\right)\tau -\pi \frac{N-2}{N}k_{b}$$

According to the displacement formula Δ*R* = *c*Δτ/2, the target displacement can be obtained:

(21) $$\Delta R=\frac{c}{4\pi \left(f_{0}+\frac{N-2}{N}\frac{B}{2}\right)}\Delta \Phi_{b}$$

Notice that the displacement calculated by Equation (21) is the same as that of microwave interferometry reported in [18,21], and the accuracy of Equation (21) can reach sub-millimeter level since the initial frequency *f_o_* of transmitted microwaves is usually at least several GHz. However, the unambiguous range is less than λ/2, which indicates that the displacement can only be obtained exactly when the actual displacement among successive samplings does not exceed λ/2. In addition, the measurement speed, a key parameter for dynamic displacement measurement, should also be noted. Supposing that the repeating frequency of transmitted microwave is 100 Hz, *i.e.*, 1/*T* = 100 Hz, the maximum measurement speed will be 100 Hz. If the time spent on data acquisition, processing and storing is excluded, a measurement speed of 10 Hz can be achieved. In actual civil infrastructures, the resolution of displacement measurement is usually at the mm level with a measuring rate of less than Hz level, so the displacement monitoring of infrastructures can be realized using Equation (21). Moreover, the integral deformation and structural modal behavior of infrastructures can also be obtained by installing multiple targets on the structure.

It should be noted that the accurate measurement using Equation (21) is guaranteed by the phase synchronism among successive samplings. As shown in Figure 1, the DAC and ADC could be contained in the same unit, and the starting time of ramp generation and signal sampling can be exactly controlled by the program. Therefore, the phase synchronism can be guaranteed by a precise oscillator and proper programming.

## 4. Displacement Measurement of Multiple Targets

### 4.1. Measurement Principle of Multiple Targets

The maximum number of the targets distinguished by the FMCW radar sensor is determined by the largest target distance *R*_max_ and the range resolution Δ*R_res_*:

(22) $$Num_{\max}=R_{\max}/\Delta R_{res}$$

**Figure 5:**
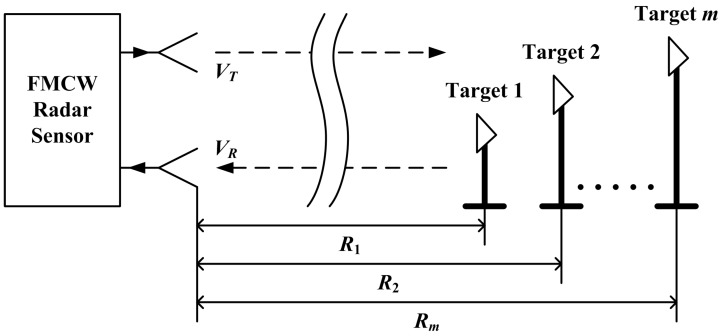
The measuring scheme for multiple targets based on FMCW radar sensor.

As long as the number of measuring targets satisfies Equation (22), all these targets could be measured by the FMCW radar simultaneously. Supposing that there are *m* targets located at the front of radar sensor as shown in Figure 5, the received signal will be the superposition of *m* terms of the form of Equation (15):

(23) $$\begin{array}{cc} s_{bM}\left(n\right)=\sum_{i=1}^{m}A_{i}e^{j\left(4\pi B\tau_{i}/N\cdot n+2\pi f_{o}\cdot \tau_{i}\right)}, & 0\leq n<N/2 \end{array}$$

where *A_i_* is the amplitude of the beat signal of the *i*th target, and τ*_i_* is the time delay induced by the corresponding target [26]. We can obtain the spectrum of the superposition signal by applying DFT to Equation (23):

(24) $$S_{B}\left(k\right)=\sum_{i=1}^{m}\begin{array}{cc} A_{i}\exp j\left[2\pi \left(f_{0}+\frac{N-2}{N}\frac{B}{2}\right)\tau_{i}-\pi \frac{N-2}{N}k\right]\cdot \frac{\sin\left[\pi \left(B\tau_{i}-k\right)\right]}{\sin\left[\frac{\pi }{N/2}\left(B\tau_{i}-k\right)\right]}, & 0\leq k<N/2 \end{array}$$

According to the spectrum of Equation (24) and range resolution of Equation (18), it can be concluded that the targets can be separated from each other while not in the same range bin. However, the target displacement may be influenced by other targets due to the spectrum leakage. Supposing that the corresponding frequency bin of first target is *k*_1_, the phase at the bin *k*_1_ will not be the one expressed by Equation (20) and it should be expressed as:

(25) $$\Phi_{1}=\arctan\left(\frac{Y_{1}+\sum_{i=2}^{m}y_{i}}{X_{1}+\sum_{i=2}^{m}x_{i}}\right)$$

where:

$$\left\{ \begin{array}{l} X_{1}=A_{1}\cos\left[2\pi \left(f_{0}+\frac{N-2}{N}\frac{B}{2}\right)\tau_{1}-\pi \frac{N-2}{N}k_{1}\right]\cdot \frac{\sin\left[\pi \left(B\tau_{1}-k_{1}\right)\right]}{\sin\left[\frac{\pi }{N/2}\left(B\tau_{1}-k_{1}\right)\right]}\\ Y_{1}=A_{1}\sin\left[2\pi \left(f_{0}+\frac{N-2}{N}\frac{B}{2}\right)\tau_{1}-\pi \frac{N-2}{N}k_{1}\right]\cdot \frac{\sin\left[\pi \left(B\tau_{1}-k_{1}\right)\right]}{\sin\left[\frac{\pi }{N/2}\left(B\tau_{1}-k_{1}\right)\right]}\\ x_{i}=A_{i}\cos\left[2\pi \left(f_{0}+\frac{N-2}{N}\frac{B}{2}\right)\tau_{i}-\pi \frac{N-2}{N}k_{1}\right]\cdot \frac{\sin\left[\pi \left(B\tau_{i}-k_{1}\right)\right]}{\sin\left[\frac{\pi }{N/2}\left(B\tau_{i}-k_{1}\right)\right]}\\ y_{i}=A_{i}\sin\left[2\pi \left(f_{0}+\frac{N-2}{N}\frac{B}{2}\right)\tau_{i}-\pi \frac{N-2}{N}k_{1}\right]\cdot \frac{\sin\left[\pi \left(B\tau_{i}-k_{1}\right)\right]}{\sin\left[\frac{\pi }{N/2}\left(B\tau_{i}-k_{1}\right)\right]} \end{array}\right.$$

According to the phase demodulation Equation (25), the phase of the first target can be obtained through the spectrum at the frequency bin *k*_1_. However, except the spectrum of the first target, the spectrum at the frequency bin *k*_1_ also contains redundant spectrums induced by the spectrum leakage of the other targets. It is natural to come up with that for multi-target displacement measurement, each target will be affected by the others. To further analyze the effect between these targets, a simulation with three targets is carried out.

### 4.2. Simulation Analysis of Displacement Measurement of Multiple Targets

**Figure 6:**
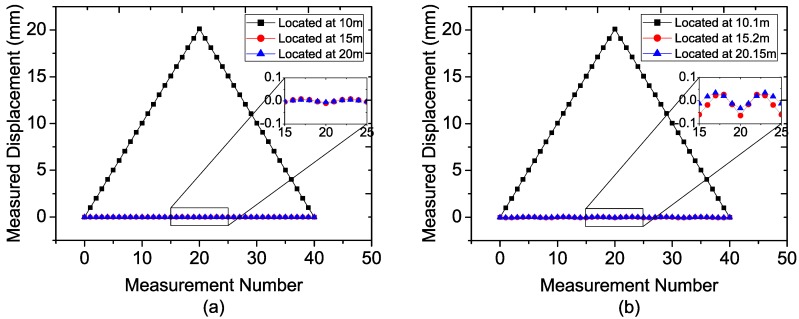
Simulation results for (**a**) Case 1; (**b**) Case 2.

The radar parameters, listed in Table 2, are chosen in accordance with the actual radar parameters used in our prototype. Two cases with different target locations are simulated as listed in Table 3. The first one is that the three targets are located just at the center of the range bins of 10 m, 15 m, and 20 m, indicating that spectrum leakage will scarcely appear. And another is that the targets are located at the distance of 10.1 m, 15.2 m, and 20.15 m, which is more in line with the actual application. Notice that both cases satisfy the range resolution of 0.5 m calculated by Equation (18). During the simulation, the target in the range bin of 10 m is moved while the other two keep still. Firstly, the target is moved backward with the step of 1 mm until the total displacement achieves 20 mm, then forward with the same step to the initial point. The simulation results are shown in Figure 6.

As can be seen from Figure 6, the displacements of target 1 can be demodulated correctly for both cases, and the two still targets in Figure 6b appear with bigger fluctuations than those in Figure 6a. Notice that the spectrum leakage of target 1 of case 2 is bigger than that of case 1, and according to the Equation (25), spectrum leakage might be used to explain the fluctuations. Although the target displacements are affected by each other, the fluctuation is less than 0.1 mm that could satisfy the requirement of millimeter level accuracy [5]. Therefore, the FMCW radar sensor is available for the accurate displacement measurement of multiple targets.

**Table 2:** Radar parameters.

Parameters	Initial Frequency *f_o_*	Bandwidth *B*	Period of Triangle Wave *T*	Sampling Rate *f_s_*
Value	24 GHz	300 MHz	0.01 s	200 kHz

**Table 3:** Target distances for simulation.

		Target 1	Target 2	Target 3
	
Case 1		10 m	15 m	20 m
Case 2		10.1 m	15.2 m	20.15 m

## 5. Prototype of a FMCW Radar Sensor

As shown in Figure 3, the whole radar sensor contains a great many components and might be time-consuming to manufacture. In order to verify the feasibility of the FMCW radar for multi-target displacement measurement in a short time, an off-the-shelf radar frontend and DAQ card are chosen for the realization of a FMCW radar prototype.

### 5.1. Main Components of the Radar

The radar frontend IVS-179 (InnoSenT, Donnersdorf, Germany), consisting of integrated VCO, transmit/receive antennas, mixers and IF-amplifiers, has the capabilities of generating, transmitting, receiving microwave and demodulating beat signals, and thus is chosen for the development of our FMCW radar sensor. The sweep frequency of the radar is from 24 GHz to 24.3 GHz, as listed in Table 1, and it is controlled through the *V_tune_* pine of the radar frontend. The maximum output power of the radar frontend is 20 dBm and split transmit/receive path is designed to increase the isolation between transmitter and receiver. The antennas are realized by 14 × 4 patch array with 7 × 28° antenna pattern and they are significantly smaller than the horn antennas with the same antenna pattern.

**Table 4:** Configuration of sampling parameters.

		Sample Rate	Buffer Size
	
DAC		400 kHz	4000
ADC		200 kHz	4000

The National Instruments (NI, Austin, TX, USA) USB 6251, containing DAC and ADC, is chosen as the DAQ card. The sampling rate and storage buffer of both DAC and ADC, used in the radar sensor, are shown in Table 4. The frequency of *V_tune_*, determined by sampling rate of DAC and storage buffer, affects the frequency of beat signal and the speed of displacement measurement, and it is set at 100 Hz. The measuring speed of the actual radar sensor could achieve about 12 Hz, which could realize the dynamic measurement at a low frequency. To display the measurement results in real time, the signal processing and data storing are done by LabVIEW in a Personal Computer (PC).

**Figure 7:**
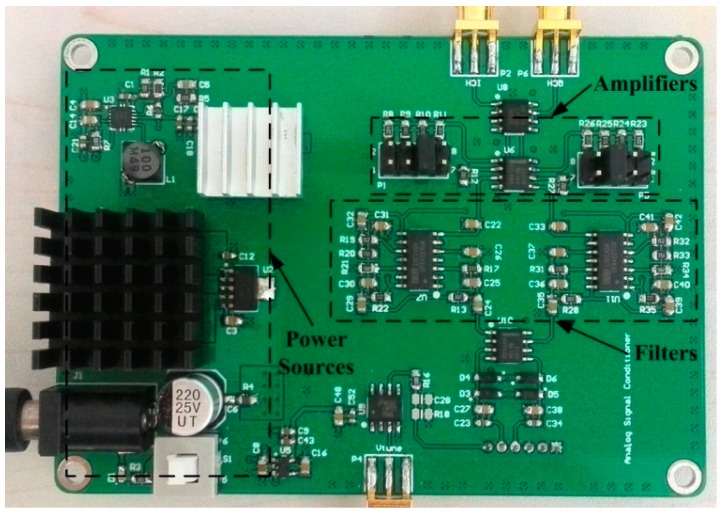
Photograph of the signal-conditioning circuit.

Between the radar front-end and DAQ card, a signal-conditioning circuit designed with proper bandwidth and variable gain is needed to remove the interference signal and amplify the beat signal. The circuit mainly contains band-pass filters and voltage amplifiers, as shown in Figure 7. The pass frequency of the filter is designed from 1 kHz to 20 kHz and the gain of the amplifier is adjustable

### 5.2. Actual Prototype Architecture

**Figure 8:**
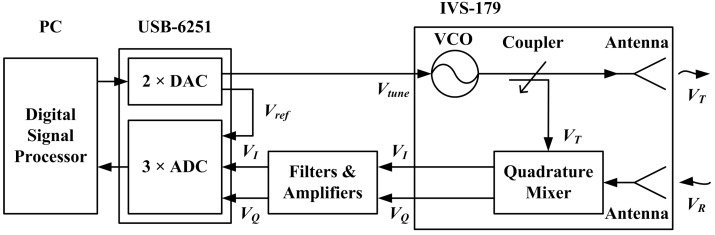
Actual architecture of the radar prototype.

**Figure 9:**
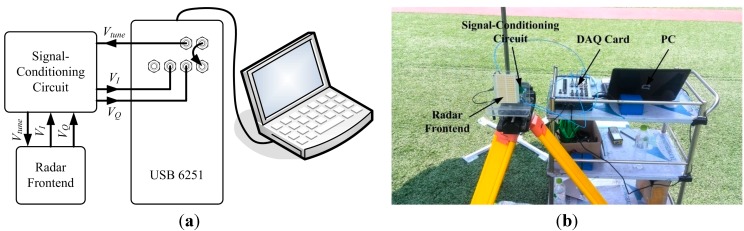
Fabricated FMCW radar sensor. (**a**) Specific connection between actual components; (**b**) Photograph of the radar.

The FMCW radar prototype can be realized by employing the above hardware. However, because the software of the DAQ card is programmed in LabVIEW, the starting time of DAC and ADC could not be exactly controlled, and the time accuracy is only about 1 ms which will seriously affect the phase synchronism. Therefore, in order to decrease the time error as much as possible, the system architecture is adjusted to be the one shown in Figure 8. The specific connection between actual components is shown in Figure 9, as well as a photograph of the radar.

**Figure 10:**
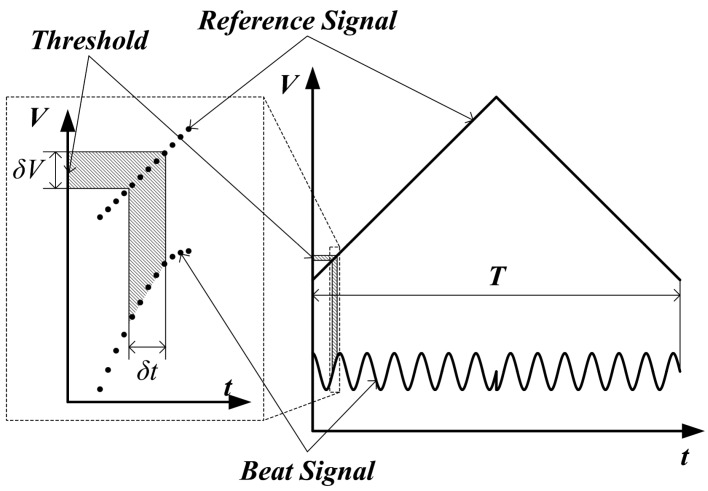
The procedure of determination of starting point.

Although the system architecture is adjusted, the phase synchronism still cannot be completely guaranteed. The selection procedure of beat signal from the sampled data is shown in Figure 10. It is easy to notice that the starting sampling time of beat signal is not precise, and the time error δ*t* is related to deviation number δ*N* included in the judgment region and sampling frequency *f_s_*:

(26) $$\delta t=\delta N/f_{s}$$

The time error will result in phase asynchronism and affect the accuracy of target displacement. It should be noticed that the phase error δΦ*_error_* induced by the time error δ*t* is also related to the beat frequency, indicating that the phase error is related to the target distance. Therefore, it is necessary to discuss whether or not the radar in Figure 9 can be employed to verify the proposed sensing method, and the displacement error induced by phase asynchronism is analyzed as follows.

Supposing that the frequency of beat signal is *f_b_*, the phase error δΦ*_error_* can be obtained:

(27) $$\delta \Phi_{error}=2\pi f_{b}\cdot \delta t$$

We can get the displacement error by combining Equations (17), (21), (26) and (27), *k_b_* = *Bτ* and *R* = *c*τ/2:

(28) $$\delta R_{error}=\frac{2B\lambda }{cTf_{s}}\delta N\cdot R$$

where $\lambda =c/\left(f_{0}+\frac{N-2}{N}\frac{B}{2}\right)$.

It can be seen that the displacement error is related with a series of parameters including bandwidth *B*, microwave wavelength λ, microwave speed *c*, sweeping period *T*, sampling rate *f_s_*, deviation number δ*N* and target distance *R*. For the radar prototype in Figure 9, all of these parameters will be fixed except *R* while displacement experiments are being performed. According to the parameters listed in Table 1 and Table 3, the target distance *R* corresponding to 1 mm displacement error can be calculated to be 40.25 m if δ*N* is set to 2, the smallest number to ensure the measurement operation. Therefore, the radar prototype in Figure 9 could be used to carry out the verification experiment of the proposed sensing method at a distance of dozens of meters.

## 6. Experiment and Discussion

Single-target and three-target displacement experiments were carried out with the fabricated radar prototype in outdoor scenarios. The single-target experiments mainly evaluate the performance of the radar prototype and verify the analysis of the displacement error in Equation (28). Since a target distance of 42 m would cause a 1 mm displacement error, the target distances in single-target experiments are chosen to be 20 m, 30 m, 40 m and 50 m, respectively. The three-target experiments are used to verify the feasibility of using the FMCW radar for multi-target displacement measurements. To decrease the phase asynchronism as much as possible, all three of the targets are located within a range of less than 30 m, and the distances are about 10 m, 15 m and 20 m.

### 6.1. Single-target Experiment Using One Corner Cube

**Figure 11:**
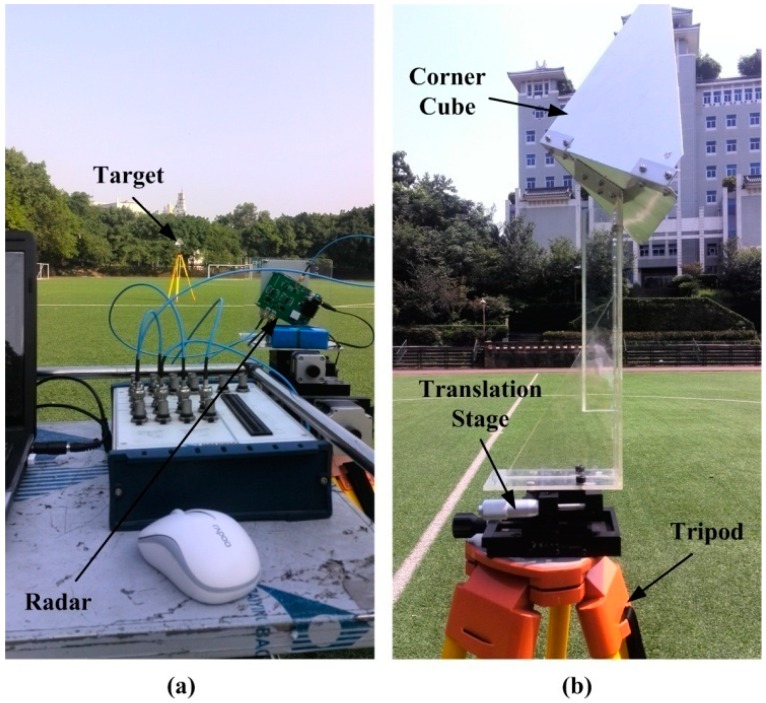
Photograph of the outdoor experiment for: (**a**) single target setup; (**b**) the target with a wooden tripod.

Figure 11a shows a photograph of the one target experiment setup. The corner cube with side length of 15 cm is fixed on a translation stage with a displacement accuracy of 10 μm, and they are placed on a wooden tripod as the target, as shown in Figure 11b. Four displacement experiments are carried out at four different target distances (20 m, 30 m, 40 m and 50 m) to test the performance of the fabricated sensor. In each experiment, the target is moved in 1 mm steps and the total displacement is 20 mm. At each step, fifty measurement results are recorded.

**Figure 12:**
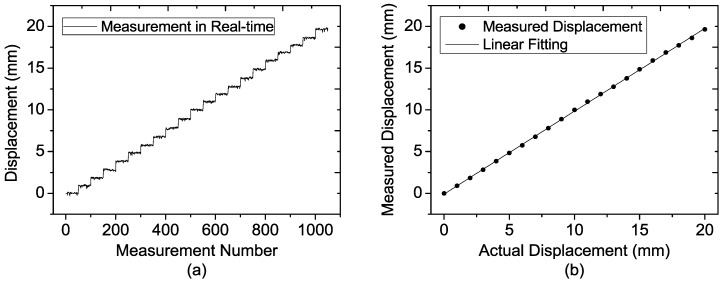
Measured results of the target located at the distance of 20 m at each step for (**a**) 50 single-measurement results; (**b**) Average of the 50 single-measurement results.

**Figure 13:**
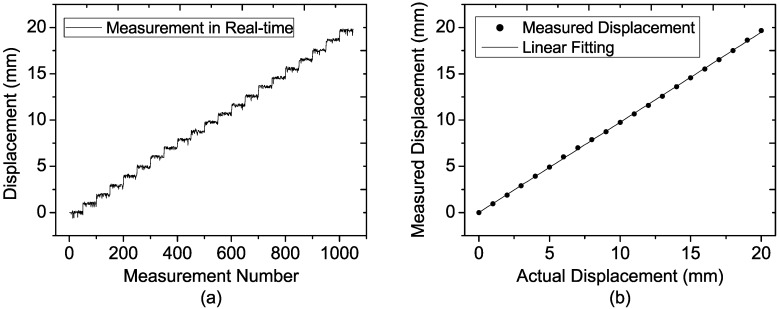
Measured results of the target located at the distance of 30 m at each step for (**a**) 50 single-measurement results; (**b**) Average of the 50 single-measurement results.

**Figure 14:**
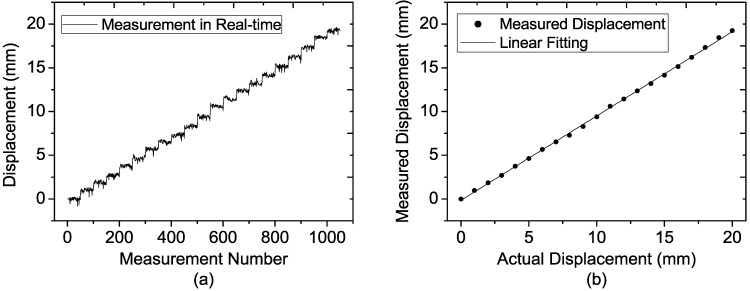
Measured results of the target located at the distance of 40 m at each step for (**a**) 50 single-measurement results; (**b**) Average of the 50 single-measurement results.

**Figure 15:**
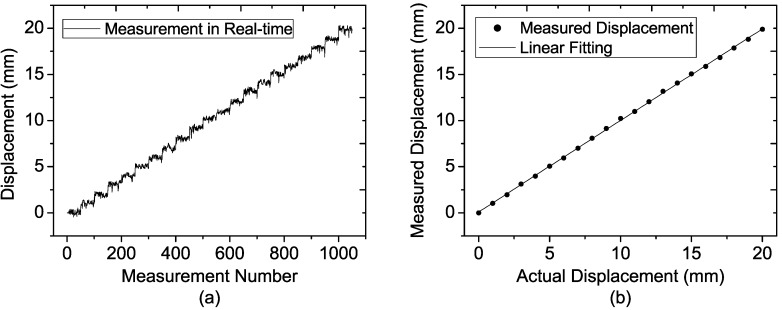
Measured results of the target located at the distance of 50 m at each step for (**a**) 50 single-measurement results; (**b**) Average of the 50 single-measurement results.

The measurement results of the four experiments are shown in Figure 12, Figure 13, Figure 14 and Figure 15, and in each figure the average of each step is depicted, as well as the fifty single-measurement results. The target movement can be clearly seen even if it is located at 50 m as depicted in Figure 15, which also indicates that a millimeter displacement accuracy could be achieved at the range of 50 m. Comparing all the results at different distances, it is easily observed that the width of the step becomes wider and wider as the distance increases. To some extent, the degradation of precision is induced by the decreasing signal to noise (SNR) and the displacement error of Equation (28).

To analyze the displacement error of the experiments, the actual measurement results at each distance are processed as follows: for each distance, (1) calculate the standard deviation of each step from the 1st one to 21st one, (2) take the average of these 21 values as the experimental precision of this distance. The theoretical value calculated by Equation (28) should be taken as an error range, *i.e.*, ±3σ [28], so the theoretical standard deviation can be estimated by δ*R_error_*/6. The standard deviation of the experimental results and theoretical calculation are given in Table 5. It can be seen that the experimental values roughly agree with the theoretical values, verifying the analysis of displacement error of Equation (28). Meanwhile, the experimental value at each distance is a little larger than the theoretical value. The reason of the difference might be the low SNR as the Radar Cross Section of the target is not changed when the distance is farther.

**Table 5:** Precision comparison of experiment results with theoretical value.

		Target Location			
		20 m	30 m	40 m	50 m
Experiment		0.091 mm	0.136 mm	0.201 mm	0.235 mm
Theory		0.08 mm	0.124 mm	0.166 mm	0.21 mm

### 6.2. Multi-Target Experiment Using Three Corner Cubes

**Figure 16:**
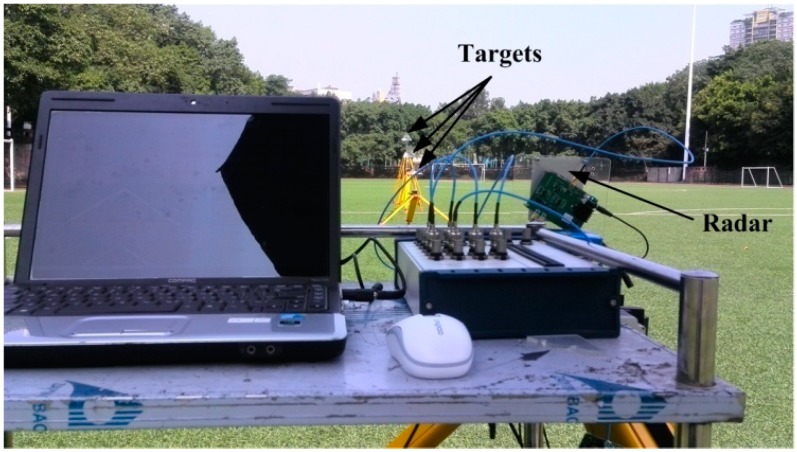
Photograph of the outdoor experiment with three targets.

Figure 16 shows a photograph of the three target experiment setup, where all the targets are the same as the one used in the single-target experiment. The three targets are placed in a line with different heights and they are located at distances of about 10 m, 15 m and 20 m, respectively. In the experiment, the movement pattern of the targets is the same as that used in the simulation of Section 4.2 and the three targets are moved one by one. In addition, a stability test of the radar sensor is carried out with all the targets keeping still. For the measurement of each target, the total time used is about 10 min.

**Figure 17:**
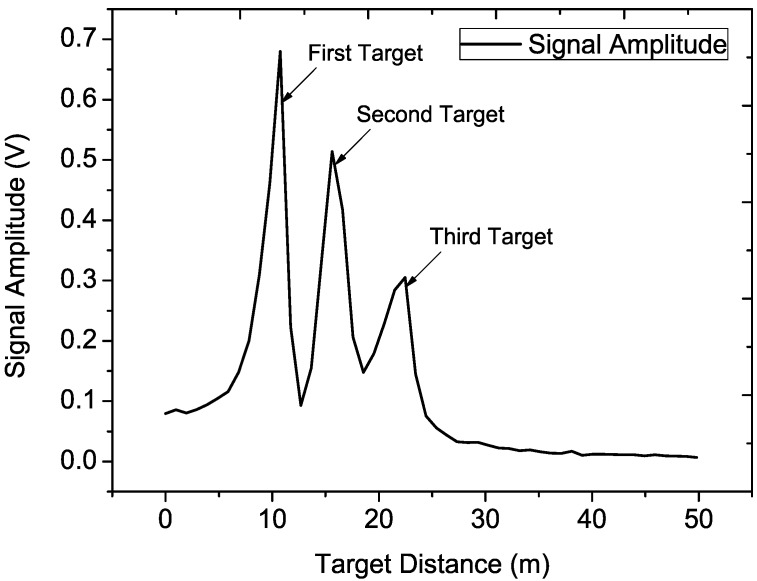
Spectrum of the beat signal in the three targets experiment.

Figure 17 depicts the spectrum of the beat signal in the three-target experiments shown in Figure 16. The three targets could be distinguished with each other clearly, and the SNR of the beat signal is high. The distance of the third target is about 22 m due to the fact that all of the targets are placed only with rough estimation. To improve the signal amplitude, the Radar Cross Section of the target might be larger while the target is located farther away.

**Figure 18:**
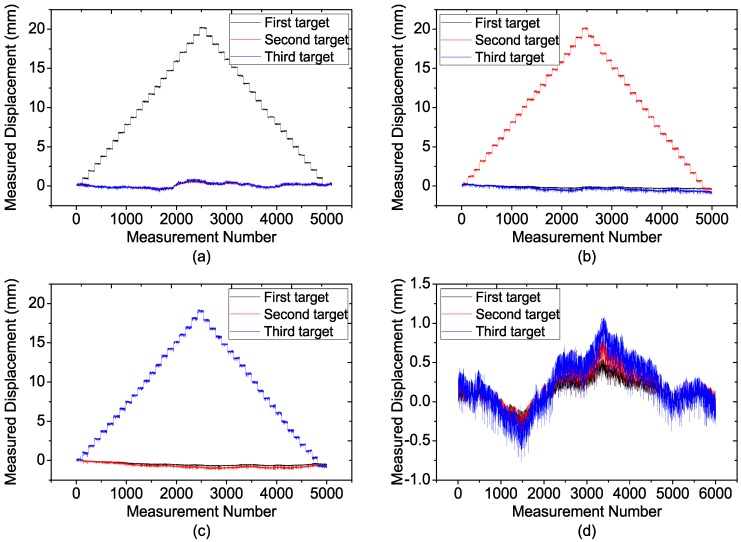
Measurement results of three targets for (**a**) Only the first target is moved; (**b**) Only the second target is moved; (**c**) Only the third target is moved; (**d**) All the targets keep still.

The displacement results of each target in the above experiments are presented in Figure 18, where the displacement of each moved target can be measured precisely. Meanwhile, we note that for all the experiments, especially those in Figure 18a,d, the measured results are not absolutely steady while the targets keep still. The fluctuation has some impact on the precise displacement measurement, and may be mainly induced by the changing temperature since it is found that the outdoor temperature was varying during the experiment. However, it still can be concluded that FMCW radar can be employed for precise multi-target displacement measurement.

## 7. Conclusions

This paper presents a usable noncontact FMCW radar sensor for multi-target displacement measurement in SHM. The measuring principle of 3-D displacement of the target on infrastructure is analyzed, which indicates that high-accuracy 3-D displacement has a direct relation with the high-accuracy measurement of 1-D distance change. Along with the purpose of simultaneous measurement for multiple targets, the FMCW radar sensor is proposed for displacement measurement in SHM. The principles of high-accuracy displacement measurement and target identification using FMCW radar are discussed specifically. To verify the feasibility of the proposed measuring method, a FMCW radar prototype was fabricated utilizing off-the-shelf devices, and the phase asynchronism induced by the hardware limitation is analyzed. Finally, outdoor experiments were carried out with one target and three targets, respectively. Experimental results show that the prototype achieves millimeter level displacement accuracy and has multi-target identification capability. Compared with the results reported in Table 1, the simultaneous displacement measurement of multiple targets at a larger distance is verified, which indicates that FMCW radar sensor could be employed for simultaneous high-accuracy multi-target displacement measurement in SHM. Although the performance of the radar prototype is not as perfect as those of interferometric radar sensors (IDS, Pisa, Italy) [3,4,15], the much cheaper FMCW radar has been verified to be potentially useful in SHM by our experimental results. Therefore, if the FMCW radar with professional signal conditioning, sampling and processing circuits is further optimized and developed, it has the potential to enrich the range of measuring sensors used in SHM.
